# Surgical safety checklist: fact or fake?

**DOI:** 10.31744/einstein_journal/2022CE0059

**Published:** 2022-09-06

**Authors:** Alexsandro Tartaglia, Marcos Antonio Almeida Matos

**Affiliations:** 1 Escola Bahiana de Medicina e Saúde Pública Salvador BA Brazil Escola Bahiana de Medicina e Saúde Pública, Salvador, BA, Brazil.; 2 Hospital Geral Roberto Santos Salvador BA Brazil Hospital Geral Roberto Santos, Salvador, BA, Brazil.

Dear Editor,

In 2008, the World Health Organization (WHO) launched the Surgical Safety Checklist as part of the Global Patient Safety Challenge to reduce surgical morbidity and mortality worldwide. The list comprises three stages: before induction of anesthesia (sign in), before skin incision (timeout), and before patient leaves operating room (sign out), which consider the regular flow of surgical procedures.^(1)^

The checklist is a simple and cheap intervention that may avoid half of postoperative deaths.^(2)^ If the implementation is carefully performed and all those involved understand the working process in the operating room, it may result in improvements. However, despite favorable evidence, the generalized adoption of the Surgical Safety Checklist has been inconsistent.^(3)^

Like any tools or processes aimed to improve safety, an ineffective implementation would reduce the expected benefits.^(4)^ This fact was shown in an article published in 2021, in the British Medical Journal (BMJ) entitled “Timeout procedure in paediatric surgery: effective tool or lip service? A randomised prospective observational study.”^(5)^ Errors were randomly introduced into 120 surgical procedures in the timeout routine of elective pediatric procedures. Selected errors included patient's wrong name, incorrect procedure, wrong side, allergy, among others. In general, 54% of errors were reported loudly during the timeout process, and the remainder were not noticed. Thus, almost half of the errors introduced in the timeout routine were not reported by the team in that study. Wrong data were pointed out more frequently by anaesthesiologists (64%), followed by the nursing team (28%), residents (6%), and undergraduate medical students (1%). Several factors may diminish the efficacy of Surgical Safety Checklists. Stress and work process specificities in the surgical scenario, together with the hierarchical structure prevalent in operating rooms, may lead to omission of relevant data.^(5,6)^

Based on this scenario, the Surgical Safety Checklist seems not simple to be implemented. Repeating its items in an operating room and demanding its use are not enough.^(3,5)^ This instrument may easily become a passive exercise, instead of a tool of active safety.^(5)^

A checklist itself is not enough to achieve the ideal outcomes.^(4)^ The apparent simplicity of this tool does not ensure the quality and safety of its practice in the operating room.^(3–5)^

An important mechanism of action of the checklist is encouraging change in behavior at the operating room to create an atmosphere of effective communication and a culture of safety.^(3)^ If properly used, the list may indeed help changing communication patterns and working processes at the operating room. A well-consolidated Surgical Safety Checklist will depend on good communication and team work, which is not always the situation observed in several organizations.^(6)^

The Surgical Safety Checklist might have been neglected by health professionals in hospitals all over Brazil. Hence, acknowledging this fact could be the kickoff for an urgent and necessary reflection, even if it reveals inconsistences regarding the working processes in the operating room. This is our hint!

## References

[1] World Health Organization (WHO). Implementation manual WHO Surgical Safety Checklist 2009: safe surgery saves lives. Geneva: WHO; c2009 [cited 2022 Jan 8]. Available from: http://whqlibdoc.who.int/publications/2009/9789241598590_eng.pdf

[2] Urbach DR, Dimick JB, Haynes AB, Gawande AA. Is WHO's surgical safety checklist being hyped? BMJ. 2019;366:l4700. 10.1136/bmj.l4700 31383633

[3] Weiser TG, Haynes AB. Ten years of the Surgical Safety Checklist. Br J Surg. 2018;105(8):927-29. 10.1002/bjs.10907 PMC6032919 29770959

[4] Weinger MB. Time out! Rethinking surgical safety: more than just a checklist. BMJ Qual Saf. 2021;30(8):613-17. 10.1136/bmjqs-2020-012600 33758034

[5] Muensterer OJ, Kreutz H, Poplawski A, Goedeke J. Timeout procedure in paediatric surgery: effective tool or lip service? A randomised prospective observational study. BMJ Qual Saf. 2021;30(8):622-7. 10.1136/bmjqs-2020-012001 PMC8311082 33632757

[6] Catchpole K, Russ S. The problem with checklists. BMJ Qual Saf. 2015;24(9):545-9. 10.1136/bmjqs-2015-004431 26089207

